# The Reduction of Infant Mortality

**Published:** 1916-07-08

**Authors:** 


### THE REDUCTION OF INFANT MORTALITY.

### The Priestley Lecture, by the Duchess of Marlborough.

On Thursday, June 29, at the Royal Society of Medicine, the Duchess of Marlborough delivered the Lady Priestley Memorial Lecture of the National Health Society before a large audience, included in which were Lady Duncannon, Lady Bowlby, Emily Lady Laurence, Lady Barran, Lady Roxburgh, Lady Barlow, Lady Dewar, Lady Broadbent, Sir Robert Morant, K.C.B., Bishop Boyd-Carpenter, and Sir Ray Lankester, K.C.B. The chair was occupied by Sir James Crichton-Browne, F.R.S.

The Chairman said the highly gifted woman whom this lecture commemorated was a pioneer in almost every department of national health work, and especially infant care and welfare. She studied the art of motherhood at a time when it was scarcely recognised as an art, and was associated with the late Dr. West in establishing that noble institution, the Hospital for Sick Children, Great Ormond Street. Her experience in this work opened her eyes to the mischiefs which arose from the neglect of children when their mothers were employed in industrial pursuits away from home, a condition of affairs which had now assumed unprecedented dimensions in this country. Lady Priestley opened what was probably the first crèche in London, and she rejoiced in having a room full of her little day-boarders. The audience would agree that this lady was an early apostle of child salvage, a burning and shining light in the dark days of pediatrics, and it was but fitting that the lecture to-day should be delivered by the gracious lady on whom Lady Priestley's mantle had evidently fallen, for her Grace had zealously participated in the movement going forward for the rescue and sustentation of child life.

The Duchess of Marlborough said that the Christian creed, on which our social institutions are based, the child's moral right to life, and the claims of Empire all constituted urgent reasons for the reduction of infant mortality. For the present high mortality of infants and young children ignorance and public neglect were equally answerable. The investigation of causes and the finding or suggesting of remedies was a medical question: it only became an administrative one when legislation was put forward in the direction of prevention. Our birth-rate was a declining one, and of a potential birth-rate of about 995,000 it was computed that 320,000 babies were lost, either in the pre-natal period or before life had lasted five years. As the heaviest toll was taken in the early weeks, it was obvious that the best results would be likely to ensue from concentrating attention on the mothers during the last weeks of pregnancy, and on the infants during their first months of life; 76 per cent. of the present infant mortality was regarded as preventible, and could be largely decreased by ante-natal care. The principal post-natal causes of infantile mortality were immaturity and congenital defects, epidemic diarrhœa and enteritis, and respiratory diseases. The death-rate was heavier among male than among female infants, among illegitimate than among legitimate, and among town than among country children. It was of great interest to reflect that had the birth-rate of 1876 held good, there would have been, in 1914, 1,346,719 births, whereas the actual number for that year was 878,000. The question of the notification of pregnancy was a very debateable one, fringing almost on the liberty of the subject; some less drastic means must be found to enable health visitors to get into touch with expectant mothers, so that medical attention could be assured for those whose confinements were liable to be complicated and difficult, especially as the number of untrained women practising midwifery was, even now, equal to the number of the trained. Moreover, a large, though variable, number of stillbirths still remained unnotified. Malnutrition and poor physique of parents was at the root of much of the child's disability. There should be a greater care of the mother, from puberty onwards, chiefly in the direction of preventing undue fatigue, exhaustion, and disease. Instruction must be greatly extended. Especially should the expectant mother be provided with nourishing meals and be guarded against fatigue, so that she should have a reserve of nourishment for her baby. There should also be an extension of the various agencies, such as crèches, schools for mothers, infant welfare centres, improved sanitary environment, and the provision of a sound food and milk supply. The lecturer reminded the meeting that probably half the ante-natal mortality of infants was ascribable to syphilis, and that in addition it was responsible for a large number of damaged lives. The intemperance associated with poverty was still—though declining—a potent cause of infant mortality, and she spoke of its effect in producing idiocy, epilepsy, and other diseases, as well as the lowering of the resistance of the body to disease generally. The place of recreation in the life of the people must be recognised, and it must be catered for in some other way than in the public-house. Great importance was attached to the more extensive provision of pre-maternity wards in hospitals, and ensuring that the working mother should, when expectant, have a much longer exemption from work than was now the case. The training of midwives should be less expensive, and they should be paid an adequate wage. Personal hygiene, mothercraft, and domestic science should be inculcated into every girl. There were now 750 infant welfare centres, most of them voluntary; some had been initiated by local authorities. Bearing in mind that prevention was always better than cure, there was a great need for workers in all branches of infant welfare; and it was of little use to tell mothers, especially poor mothers, how to protect their children from disease unless the municipal authorities provided the sanitary requirements. And there was a great need for the appointment of more women sanitary inspectors. In conclusion the Duchess suggested, amid applause, that the establishment of a National Institute of Mothercraft would constitute a fitting memorial to those who had fallen in the war; it would do much to ensure the survival of the race, and from it would radiate such educational and beneficent influences as would uplift and ennoble our conception of the status of motherhood, and in that way ensure a healthy and regenerate nation.

Bishop Boyd-Carpenter, in proposing a cordial vote of thanks to her Grace, pleaded for a wider conception of the sanctity of motherhood. Surely it was in the home that the strength, the vitality, the character of a nation was laid, and this greater reverence for maternity would shield the mother from many of the disabilities so many now suffered.

Sir E. Ray Lankester, K.C.B., seconded, and paid a high tribute to the lecturer for a thorough and unflinching examination and exposition of a great human problem.

Lady Plunkett supported the resolution, briefly mentioning her work in New Zealand in conjunction with Dr. King, and it was enthusiastically carried.

The chairman was thanked for presiding, on the motion of Mrs. Dugdale (a daughter of Lady Priestley), seconded by Dr. Amand Routh, who laid particular emphasis on the need for expectant mothers to get into touch with medical men, so that confinement difficulties could be anticipated and dealt with, especially those which would need surgical skill. There were many strong objections to compulsory notification of pregnancy, and he was convinced that some other means of ensuring due medical skill must be found.

